# Open-Flap Versus Minimally Invasive Flapless Techniques in Esthetic Crown Lengthening: A Systematic Review and Meta-Analysis of Randomized Controlled Trials

**DOI:** 10.7759/cureus.95150

**Published:** 2025-10-22

**Authors:** Mohammad D Oqlah, Husain Dashti, Lolwah F Aldhafairi, Sayed A Alsaleh, Hanan A Alshammari, Abdullah A Alenezi, Abdullah Aldulaimi, Ahmad S Shanat, Ali A Alqallaf, Abdulrahman Alsubaiei, Fahad Alhamadi, Abdulaziz Owayed

**Affiliations:** 1 Department of Dentistry, Al-Siddeeq Polyclinic, Ministry of Health, Kuwait City, KWT; 2 Department of Dentistry, Adan Specialized Center Polyclinic, Ministry of Health, Kuwait City, KWT; 3 Department of Dentistry, Andalus Polyclinic, Ministry of Health, Kuwait City, KWT; 4 Department of Dentistry, Al-Adan Polyclinic, Ministry of Health, Kuwait City, KWT; 5 Department of Dentistry, Qasr Polyclinic, Ministry of Health, Kuwait City, KWT; 6 Department of Dentistry, Ministry of Health, Kuwait City, KWT; 7 Department of Dentistry, Farwaniyah Polyclinic, Ministry of Health, Kuwait City, KWT; 8 Department of Dentistry, Al-Qurain Polyclinic, Ministry of Health, Kuwait City, KWT; 9 Department of Dentistry, Rumaithya Polyclinic, Ministry of Health, Kuwait City, KWT; 10 Division of Dentistry, School of Medical Sciences, University of Manchester, Manchester, GBR

**Keywords:** ecl, egd, esthetic crown lengthening, excessive gingival display, fl, flapless, meta-analysis, of, open-flap

## Abstract

Excessive gingival display (EGD) represents a high negative effect on the esthetic appearance. Esthetic crown lengthening (ECL) surgery is the cornerstone for the treatment of the gingival smile. However, whether to proceed with open-flap (OF) or minimally invasive flapless (FL) techniques remains unclear. We aimed in this systematic review and meta-analysis to compare the clinical outcomes between OF and FL in ECL surgeries. We searched PubMed, Cochrane CENTRAL, Scopus, and Web of Science (WOS) for randomized controlled trials including EGD patients undergoing ECL surgery with either OF or FL techniques. The primary outcome was the mean change of relative gingival margin (rGM), while the secondary outcomes were the mean changes in bleeding on probing (BOP), probing depth (PD), relative clinical attachment level (rCAL), and keratinized gingiva height (KGH). Continuous outcomes were assessed using standardized mean difference (SMD) with 95% confidence intervals (CIs) in a random-effects model. STATA 19MP (StataCorp LLC, College Station, TX) was used to pool all the statistical analyses. We included five RCTs with 180 patients in the analysis. Minimally invasive FL technique was associated with a significant reduction of rGM (SMD = -0.38; 95% CI -0.58 to -0.19, *P* < 0.001; *I*^2^ = 0%) compared to the conventional OF technique. Additionally, minimally invasive FL was associated with a significant reduction in BOP (SMD = -0.63, 95% CI -1.24 to -0.03, *P* = 0.04; *I*^2^= 73.31%) compared to the conventional OF technique. There were no significant differences regarding other studied outcomes. Minimally invasive FL and OF showed comparable results; however, the FL technique was associated with unfavorable clinical results of rGM for the treatment of EGD. Further large-volume RCTs are warranted to validate the findings.

## Introduction and background

A harmonious and aesthetic smile is an important component of facial attractiveness and can significantly boost an individual's self-esteem and social interactions. Excessive gingival display (EGD), commonly called a gummy smile, is one of the concerns that detracts from an ideal smile [1]. EGD is characterized by an abnormally exposed high amount of gingival tissue upon smiling, which can be attributed to several causes, such as vertical maxillary excess, inadequate musculature of the upper lip, or, most commonly, altered passive eruption (APE) [1]. APE is a discrepancy in the ratio between the teeth, gums, and the alveolar bone, due to failed apical migration of the gingival margin (GM) to its proper position on the cementoenamel junction (CEJ) during tooth development, thus leading to short crowns and a disproportionate smile [2]. Additionally, in APE, the gum tissue doesn't recede properly following tooth eruption, resulting in more tooth being covered with gum [2]. 

Esthetic crown lengthening (ECL) is a common treatment modality for EGD associated with APE to achieve a more balanced and aesthetic smile. Several factors, such as buccal alveolar crest location, the width of keratinized gingiva, GMs, and the mucogingival junction location, help determine the ECL approach [3]. The conventional open-flap (OF) approach aims to re-establish a normal biological width by making incisions to lift a full-thickness mucoperiosteal flap and providing direct access to the alveolar bone for recontouring [4]. During the conventional OF approach, a variety of hand or rotary tools are used for incising tissue, elevating the flap, debriding the root surfaces, and reshaping bone, which might cause injuries, including thermal or physical damage to periodontal tissue, bone, and blood vessels, particularly in cases with difficult access to the surgical site. These drawbacks resulted in greater postoperative morbidity, including increased pain, swelling, and longer periods of healing [5]. 

With the broader shift towards minimally invasive concepts in periodontal therapy, the flapless (FL) approach for ECL has emerged as a promising alternative [6]. The FL approach is linked with the use of special instruments to perform the procedure through a small hole in the gum tissue, eliminating the need for incisions and stitches. While achieving comparable aesthetic effects to the conventional OF approach, the FL technique studies suggest it may reduce the surgical time, limit the postoperative patient discomfort, and promote faster healing [7]. However, the stability and effectiveness of the FL approach, specifically in comparison to a well-documented OF approach, necessitate a robust scientific evaluation.

This systematic review and meta-analysis of RCTs aimed to evaluate the published evidence and synthesize robust conclusions regarding the conventional OF and FL approaches for ECL in the treatment of EGD associated with APE, to support more patient-centered clinical decision-making.

## Review

Methodology

We conducted this systematic review and meta-analysis according to the Preferred Reporting Items for Systematic Reviews and Meta-Analysis (PRISMA) [8], and we followed the Cochrane Handbook of Systematic Review and meta-analysis of Interventions guidelines [9].

Literature Search and Screening

We conducted an inclusive search on PubMed, Scopus, Cochrane CENTRAL, and Web of Science (WOS) from inspection until September 2025 for relevant randomized controlled trials (RCTs) without time restrictions, using the following search keywords: (("crown lengthening" OR "gingivectomy") AND ("gummy smile" OR "gingival display") AND ("flapless" OR "minimally invasive" OR "open flap" OR "conventional flap")). The search strategy was adjusted according to each database; a detailed search strategy is presented in Table 1. Furthermore, we performed backward citation analysis to ensure that we included all relevant articles. 

**Table 1:** Detailed search strategy for each database.

Database	Search terms	Search field	Search results
PubMed	(("crown lengthening" OR "gingivectomy" OR "gingivoplasty" OR "ostectomy") AND ("gummy smile" OR "gingival display" OR "altered passive eruption") AND ("flapless" OR "minimally invasive" OR "tunneling" OR "open flap" OR "conventional flap"))	All Field, English	19
Cochrane CENTRAL	(("crown lengthening" OR "gingivectomy" OR "gingivoplasty" OR "ostectomy") AND ("gummy smile" OR "gingival display" OR "altered passive eruption") AND ("flapless" OR "minimally invasive" OR "tunneling" OR "open flap" OR "conventional flap"))	Title, Abstract, Keywords, English	8
Web of Science	(("crown lengthening" OR "gingivectomy" OR "gingivoplasty" OR "ostectomy") AND ("gummy smile" OR "gingival display" OR "altered passive eruption") AND ("flapless" OR "minimally invasive" OR "tunneling" OR "open flap" OR "conventional flap"))	All Field, English	14
Scopus	(("crown lengthening" OR "gingivectomy" OR "gingivoplasty" OR "ostectomy") AND ("gummy smile" OR "gingival display" OR "altered passive eruption") AND ("flapless" OR "minimally invasive" OR "tunneling" OR "open flap" OR "conventional flap"))	Title, Abstract, Keywords, English	22

We followed a two-step process for screening citations. First, we screened the titles and abstracts of all exported citations, followed by a full-text review of eligible studies to determine whether they met our inclusion criteria.

Eligibility Criteria and Outcomes

We included only RCTs that: (1) enrolled patients with excessive gingival display who underwent esthetic crown lengthening (ECL) surgery, (2) used minimally invasive flapless (FL) techniques as the intervention, (3) used (OF techniques as the control, and (4) reported the outcomes of interest. We excluded single-arm trials, abstracts, unpublished data, and studies published in languages other than English.

Our primary outcome of interest was the mean change of relative GM (rGM), defined as the distance from a fixed point in the stent to the highest cervical point of the GM, from the baseline [10]. The secondary outcomes were the mean changes of relative clinical attachment level (rCAL), defined as the distance from the fixed point in the stent to the deepest point of GM, bleeding on probing (BoP), probing depth (PD), and keratinized gingiva height (KGH).

Quality Assessment

We used the Cochrane Risk of Bias 2 (ROB-2) tool to assess the quality and potential bias of the included studies [11]. The tool assesses five domains: (1) bias arising from the randomization process, (2) deviations from the intended interventions, (3) missing outcome data, (4) bias in the measurement of outcomes, and (5) bias in the selection of the reported results. Each domain was assessed by answering signaling questions and then classified as low-risk, some concerns, or high-risk judgment, with an overall risk of bias. Any disagreements were resolved through discussion among the authors.

Data Extraction and Statistical Analysis

We used a standardized Excel sheet to extract all the necessary data from the included studies, and it includes: (1) baseline characteristics of the patients included in each study; sample size, age, gender, plaque index, PD, and BoP, (2) summary characteristics of the included study; country, time of the study, patients characteristics, inclusion and exclusion criteria, intervention, control, outcomes assessed, key findings, and follow-up, (3) the ROB-2 domains, and (4) the outcomes data.

Continuous data were extracted as mean change from the baseline, standard deviations (SDs), and the total number of included patients. They were pooled as standardized mean difference (SMD) with its 95% confidence intervals (CIs) using a random-effect model. The heterogeneity was assessed using the Cochrane Q test, and the *I*^2^ measure was determined across all the studies. A significant heterogeneity among the studies was determined when a *P*-value less than 0.05 and *I*^2^ ≥ 50%. STATA 19MP software (StataCorp LLC, College Station, TX) was used to conduct all the analyses using “meta esize” and "meta forest plot” packages.

Results

Literature Search

**Figure 1:**
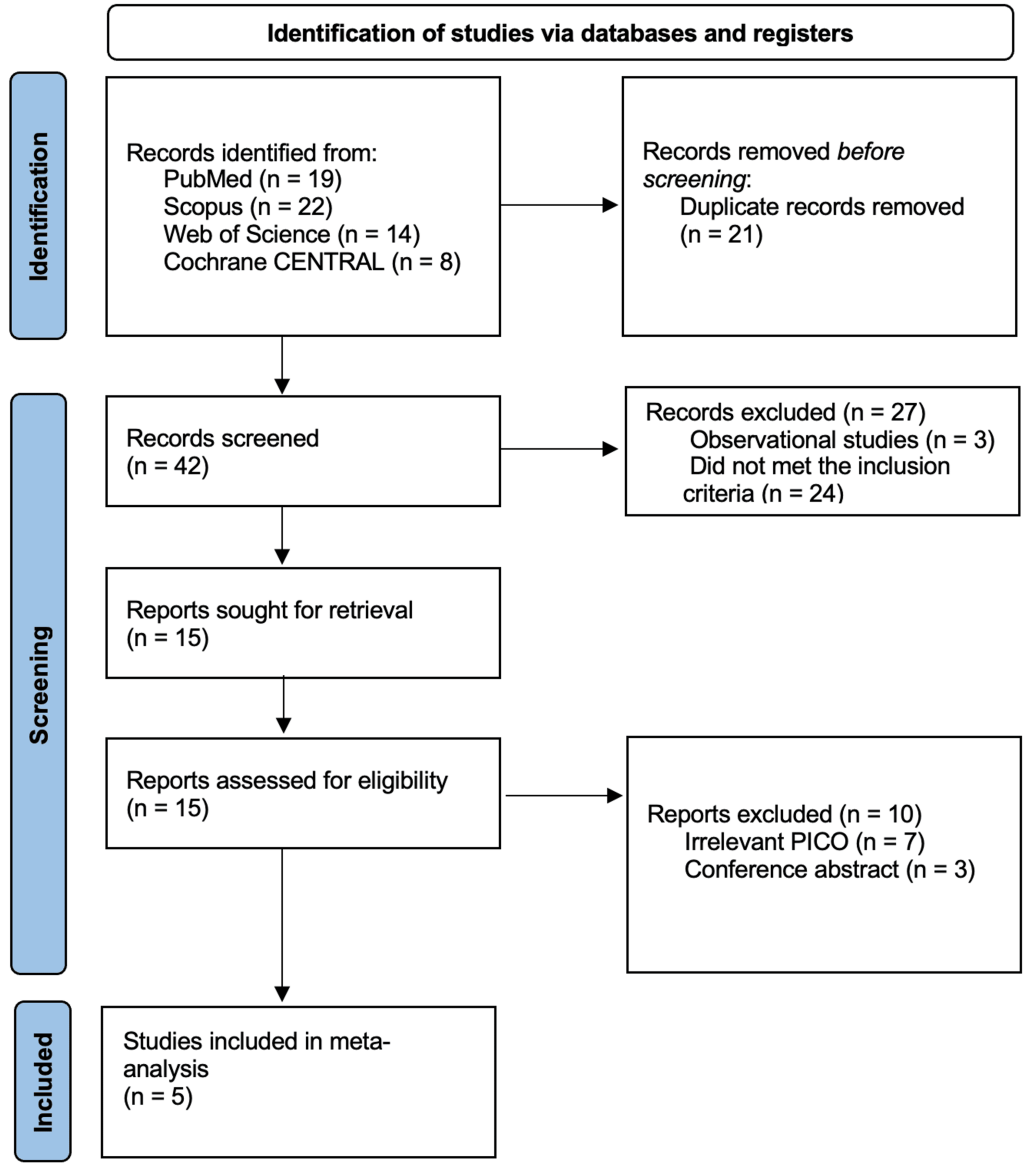
The PRISMA flow diagram. PRISMA, Preferred Reporting Items for Systematic Reviews and Meta-Analysis

Searching the different databases yielded 63 articles; a total of 21 were duplicates and removed, leaving 42 articles for the titles and abstracts screening. Only 15 articles were eligible for full-text screening; of them, 5 RCTs were finally included in the meta-analysis [12-16]. The selection process is presented in the PRISMA flow diagram (Figure 1).

Patients’ Characteristics and Risk of Bias Assessment

The five RCTs included 180 patients: 90 (50%) in the intervention group, and 90 (50%) in the control group. Of them, 80 (44%) patients were females, and the mean age ranged from 24.4 to 32 years. Detailed baseline characteristics of the patients and a summary of the included studies are presented in Tables 2-3.

**Table 2:** Summary of the included studies. APE, altered passive eruption; VAS, visual analog scale; CEJ, cemento-enamel junction; EGD, excessive gingival display; VRS, visual rating scale; rGM, relative gingival margin; PES, pink esthetic score; PI, plaque index; BoP, bleeding on probing; CAL, clinical attachment level; PD, probing depth; WKT, width of keratinized tissue; RCAL, relative clinical attachment level; RBL, radiographic bone level; GI, gingival index; KGH, keratinized gingival height; CBCT, cone-beam computed tomography; MB, mesio-buccal; RANKL/OPG, receptor activator of nuclear factor kappa-B ligand/osteoprotegerin; BT, bone thickness; GT, gingival thickness

Study ID	Country	Time of the study	Patients’	Inclusion criteria	Exclusion criteria	Intervention	Control	Outcomes assessed	Key findings	Follow-up
Sourour et al., 2024 [12]	Egypt	May 2020 - December 2021	Patients with APE Type 1B	>18 years, systemically healthy, APE type 1B, plaque scores <20%	Implants in the upper anterior, incisal edge attrition, smokers, pregnant	Flapless (FL): Microsurgical internal bevel and intrasulcular incisions, tunneling, piezosurgery for ostectomy (3 mm apical to CEJ)	Open-flap (OF): Full-thickness mucoperiosteal flap, piezosurgery for ostectomy (3 mm apical to CEJ), vertical mattress sutures	Primary: Pain (VAS). Secondary: Swelling (VRS), rGM, patient satisfaction, PES, PI, BoP, CAL, PD.	The FL group had significantly lower pain and swelling and showed earlier gingival margin stability.	6 months
ALsahli et al., 2021 [13]	Syria	November 2019 to December 2020	Patients with APE type 1B	>20 years, thin-to-moderate bone, APE 1B in ≥3 maxillary teeth per quadrant, no attachment loss	Smokers, systemic diseases, pregnant/breastfeeding, prostheses, orthodontic appliances	FL: Gingivectomy followed by intrasulcular incision, piezosurgery for ostectomy	OF: Full-thickness flap elevation after gingivectomy, piezosurgery for ostectomy, and the interrupted sutures.	PI, PD, BoP, WKT, RCAL, RBL, RGM, Pain (VAS).	The FL group showed significantly lower pain and BoP values	3 months
Dayoub and Yousef, 2019 [14]	Syria	May 2018 and November 2018	Patients with APE 1B	≥20 years, APE 1B in ≥3 maxillary teeth per quadrant, thin-to-moderate bone, no attachment loss.	Pregnant/lactating, smokers, orthodontics, previous surgery, prostheses, recent antibiotic use, systemic diseases.	FL: Gingivectomy and intrasulcular incision, piezosurgery for ostectomy via incisions	OF: Gingivectomy and intrasulcular incision, mucoperiosteal flap elevation, piezosurgery for ostectomy, interrupted sutures	PI, BoP, GI, PD, rCAL, rBL, rGM, KGH, CBCT (CEJ-BC, SGH), surgical time, pain (VAS), patient satisfaction (VAS).	The FL group had significantly shorter surgical time, less pain, and lower BoP and GI scores	3 months
Ribeiro et al., 2014 [15]	Brazil	January 2011 to July 2011	Patients with EGD due to APE	>21 years, ≥20 teeth, no sites with attachment loss and PD >3 mm, plaque/bleeding scores <15%	Pregnant/lactating, smokers, recent antibiotic use, previous surgery, systemic conditions, orthodontics	FL: Internal bevel and sulcular incisions, bone recontouring with microchisels via incisions	OF: Full-thickness mucoperiosteal flap, bone recontouring with surgical chisels, interrupted sutures	PI, MB, BoP, PD, rGM, rCAL, KGH, rBL, RANKL/OPG levels, CBCT (BT, GT), pain (VAS), patient satisfaction	Both techniques produced stable and similar clinical results up to 12 months. FL surgery was significantly faster.	12 months
Altayeb et al., 2022 [16]	United States	August 2014 to March 2019	Patients with APE	>22 years, EGD ≥3 mm, gingival overlap >19%, healthy periodontium	PD ≥3 mm, restorative procedures affecting mock-up, pregnant/lactating, smokers, systemic conditions	FL: Gingivectomy and ostectomy performed with an Er, Cr:YSGG laser.	OF: Apically positioned flap, ostectomy performed with an Er, Cr:YSGG laser.	Primary: Patient perceptions (anxiety, pain, swelling, bleeding, discomfort, sensitivity, medication use) via Likert scale. Overall satisfaction with surgical time.	The FL group had significantly less patient-reported anxiety, pain, swelling, bleeding, and discomfort. FL surgery was also significantly faster.	1 month

**Table 3:** Baseline variables of the included patients. NA, not available

Study ID	Arm	Sample size	Age, years		Gender (Female, *n*, %)	Plaque index (%, Score)		Probing depth (mm)		Bleeding on probing (%)	
			Mean	SD		Mean	SD	Mean	SD	Mean	SD
Sourour et al., 2024 [12]	Intervention	12	29.2	5.6	10 (83.3%)	15	3.55	1.7	0.28	13.67	2.46
	Control	12	32	5.3	8 (66.7%)	14.17	3.48	1.67	0.32	12.5	2.61
ALsahli et al, 2021 [13]	Intervention	16	26.5	1.3	9 (56.3%)	0.2	0.1	1.8	0.1	10.3	1.1
	Control	16									
Dayoub and Yousef, 2019 [14]	Intervention	16	24.4	3.5	11 (68.8%)	0.11	0.12	1.78	0.2	11.87	2.4
	Control	16				0.12	0.13	1.81	0.22	12.18	2.6
Ribeiro et al., 2014 [15]	Intervention	28	27.5	5.8	20 (71.4%)	9.1	3.7	1.4	0.5	9.3	3.5
	Control	28				8.3	4.1	1.4	0.5	8.9	3.8
Altayeb et al., 2022 [16]	Intervention	18	NA	NA	11 (61.1%)	NA	NA	NA	NA	NA	NA
	Control	18			11 (61.1%)						

**Figure 2:**
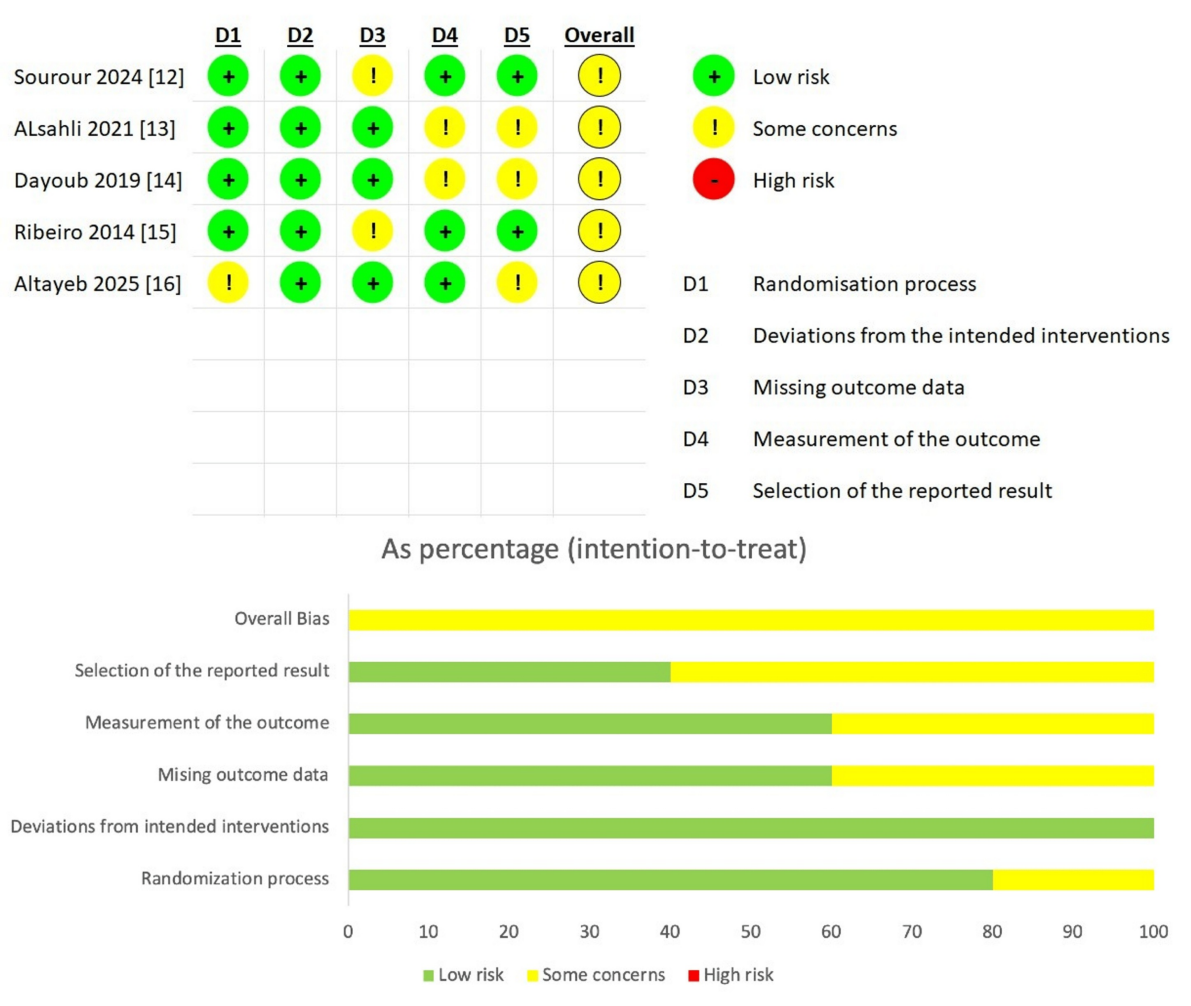
ROB assessment of the included studies. ROB, Risk of Bias

All the included RCTs were assessed using the ROB-2 tool, and all the included studies had some overall concerns, mainly in the bias arising from the measurement and the selection of the reported outcomes (Figure 2).

Outcomes

**Figure 3:**
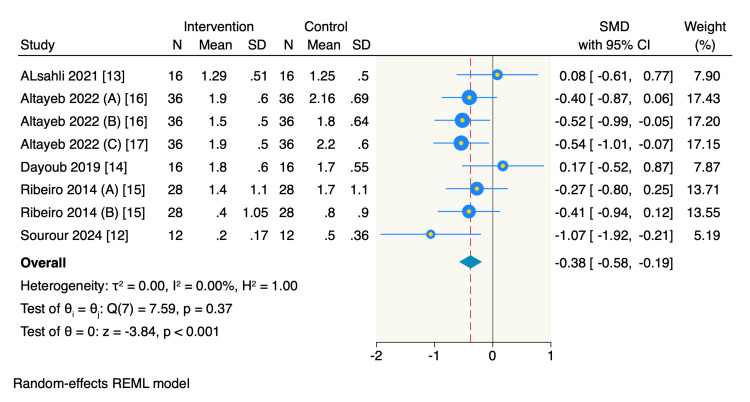
Forest plot of the mean change of rGM. Altayeb 2022 (A), rGM mean change of central incisors; Altayeb 2022 (B), rGM mean change of lateral incisors; Altayeb 2022 (C), rGM mean change of canines; Ribeiro 2014 (A), rGM mean change of mid-buccal sites; Ribeiro 2014 (B), rGM mean change of interproximal buccal sites. rGM, relative gingival margin; CI, confidence interval; SD, standard deviation

Primary outcome, rGM: All five RCTs assessed the mean change of rGM from the baseline, of which the minimally invasive FL technique showed a significant reduction in the rGM compared to the conventional FL (SMD = -0.38, 95% CI -0.58 to -0.19, *P* < 0.001; *I*^2^ = 0%) (Figure 3). 

**Figure 4:**
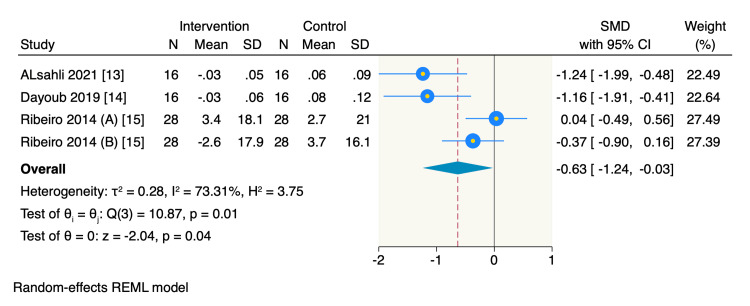
Forest plot of the mean change of BoP. Ribeiro 2014 (A), BoP mean change of mid-buccal sites; Ribeiro 2014 (B), BoP mean change of interproximal buccal sites. BoP, bleeding on probing; SMD, standardized mean difference; CI, confidence interval; SD, standard deviation

**Figure 5:**
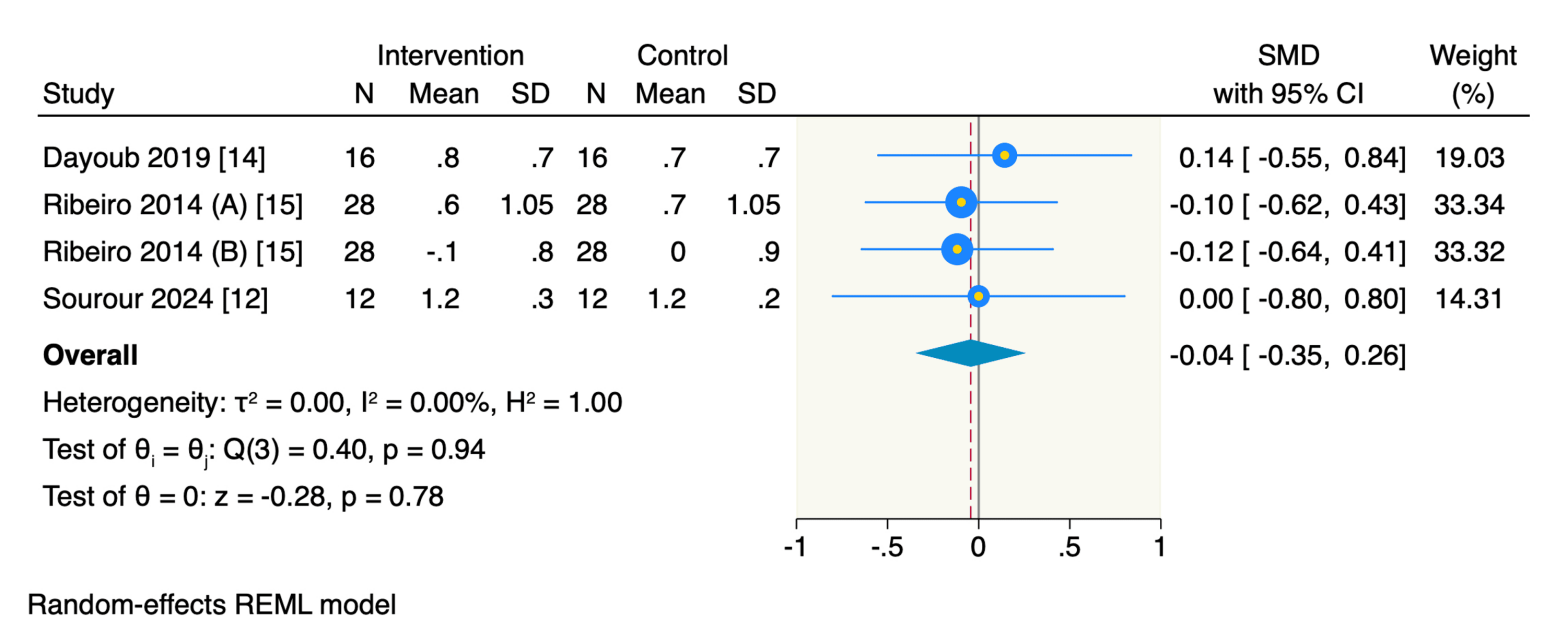
Forest plot of the mean change of rCAL. Ribeiro 2014 (A), rCAL mean change of mid-buccal sites; Ribeiro 2014 (B), rCAL mean change of interproximal buccal sites. RCAL, relative clinical attachment level; CI, confidence interval; SD, standard deviation

Secondary outcomes: Three RCTs reported the mean change in BoP from the baseline, of which the pooled estimate showed a significant decrease in BoP in the intervention group compared to the control group (SMD = -0.63, 95% CI -1.24 to -0.03, *P* = 0.04; *I*^2^ = 73.31%) (Figure 4). However, only three RCTs assessed the mean change in rCAL from the baseline, of which the pooled estimate showed no significant difference between the intervention and the control groups in decreasing the rCAL (SMD = -0.04, 95% CI -0.35 to 0.26, *P* = 0.78; *I*^2^ = 0%) (Figure 5). 

**Figure 6:**
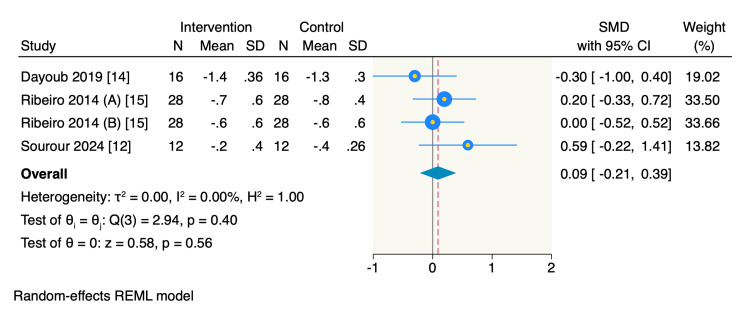
Forest plot of the mean change of PD. Ribeiro 2014 (A), PD mean change of mid-buccal sites; Ribeiro 2014 (B), PD mean change of interproximal buccal sites. PD, probing depth; CI, confidence interval; SD, standard deviation

**Figure 7:**
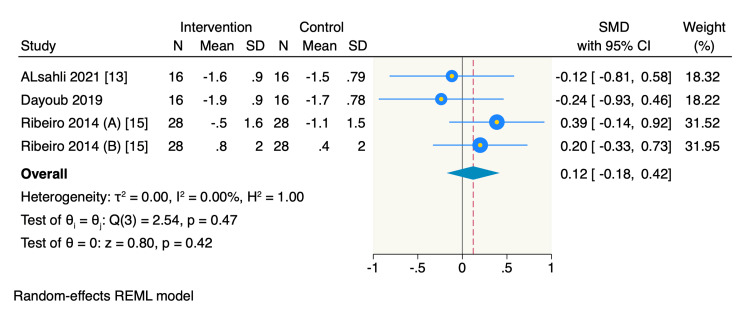
Forest plot of the mean change of KGH. Ribeiro 2014 (A), KGH mean change of mid-buccal sites; Ribeiro 2014 (B), KGH mean change of interproximal buccal sites. CI, confidence interval; SD, standard deviation; KGH, keratinized gingival height

On the other hand, only three RCTs reported the mean change in PD from the baseline, of which the pooled estimate showed no significant difference between the intervention group and the control group (SMD = 0.09, 95% CI -0.21 to 0.39, *P* = 0.56; *I*^2^ = 0%) (Figure 6). Additionally, three RCTs reported the mean change in KGH from the baseline, of which the pooled estimate showed no significant difference between the intervention group and the control group (SMD = 0.12, 95% CI -0.18 to 0.42, *P* = 0.42; *I*^2^ = 0%) (Figure 7).

Discussion

The present systematic review and meta-analysis is the most comprehensive study to assess the clinical outcomes of the conventional OF and the minimally invasive FL techniques in ECL surgeries to treat the gingival smile. Five RCTs with 180 patients were included in the final analysis, and their pooled results showed that the minimally invasive FL technique was associated with significant reductions in rGM and BoP compared to the conventional OF technique. On the other hand, there were no significant differences between the two studied techniques regarding our studied outcomes. 

There are a couple of factors that can determine whether the patient should proceed with the conventional ECL OF technique or minimally invasive FL technique, including the proportion of the attached gingiva area, and the included patients should have an adequate zone of keratinized gingival height (KGH) that would persist after the removal of the gingival tissue following the procedure [17]. Moreover, the minimally invasive FL technique is contraindicated in patients with a limited amount of KGH, because the FL technique often requires scalloped incisions that would result in mucogingival problems if there is not enough KGH; however, the OF technique often replaces the preexisting keratinized tissue in an apical position, thus allowing for keratinized tissue preservation [17]. Therefore, all the included studies adjusted for these factors while selecting patients and allocated them to the treatment groups. 

Our results showed a significant unfavorable result in the mean change in rGM from the baseline following the application of minimally invasive FL compared to the conventional OF technique; however, the two studied techniques were associated with comparable results regarding all other studied outcomes. The reduction of rGM following the FL technique compared to the OF technique could be attributed to the rebound phenomenon, which is highly characteristic of the FL raise technique. The rebound occurs as a result of the periodontium that is being reshaped in the past form during the development and maturing durations [18]. 

Additionally, all surgical procedures, including minimally invasive FL technique, were associated with higher rates of rebound phenomenon for the gingival tissue [18]. Also, the inclusion criteria of the included studies prompted the inclusion of thick phenotypes, which are highly susceptible to rebound compared to the thin phenotypes [19]. Also, the stability of the GM is often unpredictable following the ECL surgery; therefore, the inclusion of thick gingival biotype and the short distance between the margin of the flap and the buccal crest are key factors to the increased rebound phenomenon in the minimally invasive FL technique [20]. This was not aligned with a previous report of Dayoub et al. [14], in which they reported that the rebound of the tissue was observed in both groups; however, it was slightly higher in the OF group without a significant difference. Their results could be attributed to the traumatic surgical methodologies as osteotomy and flap reflection, causing more gingival tissue rebound [18].

On the other hand, the minimally invasive FL technique permitted rapid healing and less inflamed tissues compared to the OF across the included studies, and these results were supported by previous reports [13,15]. According to previous reports, most of their patients included have lower pain scores in the FL technique group compared to the OF technique, which could be attributed to the fact that the OF technique often causes an injury to the surrounding blood vessels in the periosteum [21]. Additionally, the need for surgical suturing and an increased procedure time on the OF side could result in more edema and hematoma following the surgery, thus causing severe pain [21].

Our study reported less mean change values of BoP in the FL-treated sites compared to the OF-treated sites. Moreover, our results are consistent with those of Dayoub et al. [14] and Ribeiro et al. [15], who found that the FL approach promotes faster healing and less tissue inflammation compared to the OF approach. The difference noted in their studies could be due to the tissue trauma and the increased recovery time following the flap reflection and surgical suturing, which are required in the OF technique. Also, these results could be attributed to the ability of the patients to resume a faster oral hygiene procedure in the FL-treated sites compared to the OF-treated sites. In addition to the less damage to the blood vessels following the FL techniques, all of which ensure a clear vision during the surgical procedure [22]. 

In addition, minimally invasive FL technique offers an alternative technique with critical advantages compared to the OF technique, which often requires instruments for bone resection in the ECL surgery. Moreover, the FL technique was associated with higher patient satisfaction, less surgical times in the chair, less amount of trauma due to the surgery, and accelerated healing time and patient comfort. Despite these critical advantages, the minimally invasive FL technique should be used within limitations, as this approach has numerous weakness points, like the inability to perform osteotomy on the buccal side of the alveolar bone due to the lack no visibility of the alveolar bone and other structures [23].

Limitations

Our study is the first and the most comprehensive study; however, some limitations should be addressed before generalizing the findings of the current study. First, the sample size of the included studies was relatively small, indicating the need for larger studies to further consolidate these benefits. Second, the follow-up duration among the included studies was short, with only one study reported results at 12 months of follow-up, as the amount of gingival rebound increases over longer follow-up durations, especially after six months. Therefore, further long-term studies are needed to assess the GM level changes over time and establish an optimal comparison of different surgical methods and approaches. While the studies used standardized protocols, some variations in the clinical setting were noticed, especially the initial surgical measures as plaque index, PD, and BoP of the included patients, which could be a source of heterogeneity. Finally, due to the limited number of the included studies in this meta-analysis, funnel plots and Eggar test could not be applied to investigate the potential publication bias among the included studies.

## Conclusions

Our systematic review and meta-analysis of five RCTs and 180 patients found that the minimally invasive FL technique and the conventional OF technique showed comparable results over time and are effective in ECL surgery, while accounting for the difference in the post-procedure mean values of rGM and BOP. Additionally, the FL technique appears to be an easy, minimally invasive, and predictable procedure with notable clinical advantages, with no surgical sutures, less bleeding and pain, and shorter healing times compared to the conventional OF technique.
